# Maternal and Neonatal Outcomes of Women Conceived Less Than 6 Months after First Trimester Dilation and Curettage

**DOI:** 10.3390/jcm11102767

**Published:** 2022-05-13

**Authors:** Tal Margaliot Kalifa, Eyal Lang Ben Nun, Hen Y. Sela, Fayez Khatib, Sorina Grisaru-Granovsky, Misgav Rottenstreich

**Affiliations:** 1 Department of Obstetrics & Gynecology, Shaare Zedek Medical Center, Hebrew University School of Medicine, Jerusalem 91031, Israel; taltalonm@gmail.com (T.M.K.); eyallbn@gmail.com (E.L.B.N.); dr.khatib@gmail.com (F.K.); sorina@szmc.org.il (S.G.-G.); misgavr@gmail.com (M.R.); 2 Department of Nursing, Jerusalem College of Technology, Jerusalem 93721, Israel

**Keywords:** women, cesarean delivery, complications, operative time, risk-factors, maternal outcomes

## Abstract

Objective: To evaluate the maternal and neonatal outcomes of pregnancies conceived ≤6 months after first trimester (<14 weeks) dilation and curettage (D&C). Methods: A retrospective computerized database study of women who conceived ≤6 months following a missed abortion and delivered in a single tertiary medical center between 2016 and 2021. The maternal and neonatal outcomes of women who had D&C were compared to those of women who had non-medical or spontaneous miscarriages. The primary outcome of this study was the rate of preterm birth (<37 weeks). Secondary outcomes were adverse maternal and neonatal outcomes. Univariate analysis was followed by multiple logistic regression models; adjusted odds ratios (aORs) and 95% confidence intervals (CIs) were calculated. Results: During the study period, 1773 women met the inclusion criteria; of those, 1087 (61.3%) women gave birth following D&C. We found no differences between the study groups in any maternal or neonatal parameter examined including preterm birth (PTB), miscarriage to pregnancy interval, fertility treatments, hypertension disorders of pregnancy, placental complications, mode of delivery and neonatal birth weights. This was confirmed on a multivariate analysis as well [aOR 1.74 (0.89–3.40), *p* = 0.11] for preterm birth. Conclusion: Watchful waiting or the medical treatment of a first trimester missed abortion present no more risks than D&C to pregnancies conceived within six months of the index miscarriage. Further studies in other settings to strengthen these findings are needed.

## 1. Introduction

An unfortunate obstetrical outcome occurring commonly in the first trimester is early pregnancy loss (EPL) [1,2]. Management of this situation can be done either surgically (via dilation & curettage (D&C)), medically (using a synthetic prostaglandin E1 analogue such as misoprostol) or expectantly [3,4], and the factors that may affect women’s choices include the length of hospital stay, level of pain, post procedure bleeding, recovery time, cost and chance of complications [5]. Much of the research that compared the medical/non-interventional approach to the surgical approach dealt with the question of immediate complications. While some studies found no difference in terms of complication rates, some demonstrated an advantage for D&C in lowering the risk of adverse outcomes such as incomplete abortion, the need for unplanned or additional surgical evacuation, bleeding and blood transfusion requirement [6,7,8].

Many studies that compared women with prior pregnancy loss to women without found an association between prior pregnancy loss and future adverse pregnancy outcomes including preterm premature rupture of membranes (PROM), preterm birth, intrauterine growth restriction, hypertensive disorders, preeclampsia, cesarean delivery (CD) and perinatal mortality [9,10,11]. Yet, very few studies assessed the future pregnancy outcome in regard to the specific method of uterine evacuation [12,13,14]. In a small study that assessed fertility and obstetric outcomes in women who underwent D&C or were treated with expectant management after a failed treatment with misoprostol, D&C led to similar fertility rates (conception and ongoing pregnancy rates), preterm birth rates and CD rates as expectant management did^12^. This hypothesis was challenged by a previous study which showed that, unlike the case of women who managed non-surgically, women who were treated with D&C had an increased risk of subsequent spontaneous preterm birth (PTB) compared with those with no previous pregnancy losses [15].

To provide women and clinicians with more information regarding the consequences of the mode of evacuation for the treatment of EPL on their future pregnancy, our aim was to evaluate the maternal and neonatal outcomes of pregnancies conceived ≤6 months after first trimester (<14 weeks) miscarriage, whether by D&C or by medical (using misoprostol) or expectant treatment.

## 2. Materials and Methods

### 2.1. Study Design

We conducted a single tertiary center retrospective cohort study using the computerized medical records of a university-affiliated medical center in Jerusalem, Israel—the Shaare Zedek Medical Center (SZMC)—between 2016 and 2021. Data on demographic and obstetric characteristics, as well as data on delivery complications, were extracted from the electronic database management software, which is updated during labor and validated periodically by computer systems personnel. As such, the possibility of bias inherent to retrospective studies was minimized.

The study population included all women who conceived ≤6 months following a first trimester missed or incomplete abortion and delivered between 2016 and 2021.

We included only women who conceived <6 months following the EPL and excluded those who conceived more than 6 months after, as there is some evidence to suggest that the endometrium undergoes changes that influence reproductive function up to 6 months following a first trimester D&C [16]. Additional exclusion criteria: women who had molar pregnancy, underwent a termination of a pregnancy or had two subsequent miscarriages within 6 months before conceiving.

The maternal and neonatal outcomes of women who underwent D&C were compared to those of women who underwent medical (Misoprostol) or spontaneous miscarriages.

The primary outcome of this study was the rate of preterm delivery (PTD, <37 weeks). Secondary outcomes included maternal and neonatal adverse outcomes.

Maternal morbidity parameters included: preterm labor (<37 weeks), placental abruption, mode of delivery, maternal intensive care unit (ICU) admissions, postpartum hemorrhage, blood products transfusion and disorders of the third stage of labor. Neonatal morbidity parameters included: intrauterine fetal death, neonatal birth weight, Apgar scores, neonatal asphyxia and neonatal intensive care unit (NICU) admissions.

The study was approved by the Institutional Review Board of the Shaare Zedek Medical Center (IRB approval number: 0260-21). The data were obtained from medical records and de-identified, with no direct participation of the patients. As such, written informed consent was not required.

### 2.2. Statistical Methods

The characteristics were described by proportions (nominal variables), means ± SD (continuous variables with normal distribution) and medians with interquartile ranges (IQR) (continuous variables without normal distribution), as appropriate. Categorical variables were compared using the Chi-square test or Fisher’s exact test, and continuous variables were analyzed using the unpaired Student’s T-test or the Mann–Whitney test, as appropriate.

Univariate analysis was performed to test the association between the mode of the uterine evacuation method and the different maternal demographic, obstetric and delivery characteristics. A *p*-value < 0.05 was considered statistically significant.

The variables found to be significant on the univariate analysis were included in a multivariable logistic regression, modeling the association between D&C and PTD in a subsequent pregnancy. These included gravidity and parity, previous CDs, fertility treatments, hypertensive disorders of pregnancy, multifetal gestation, diabetes (pre-gestational and gestational) and cervical dilation during the D&C.

The results of these analyses are reported as adjusted odds ratios (aOR) with 95% confidence intervals (CIs). All statistical tests were two-sided. The analyses were carried out using SPSS software (version 25 statistical package: IBM, Armonk, NY, USA).

## 3. Results

**Figure 1:**
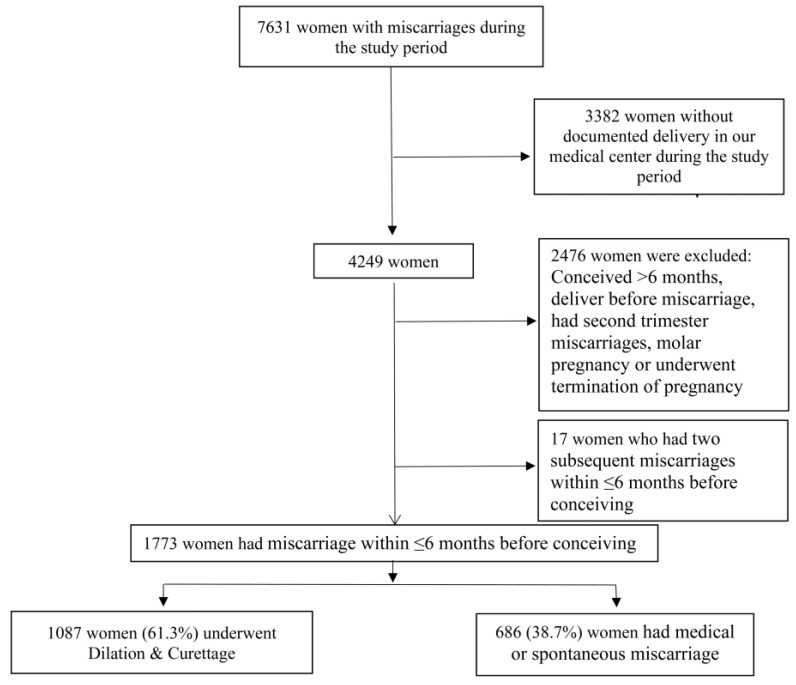
Schematic Study Flowchart.

During the study period, 1790 women conceived within 6 months following EPL. Seventeen women, of whom 9 (0.8%) underwent D&C and 8 (1.2%) had a medical or spontaneous miscarriage (*p *= 0.48), had an additional miscarriage and were excluded. Finally, 1773 women met the inclusion and exclusion criteria. Of those, 1087 (61.3%) underwent D&C due to miscarriage within 6 months prior to conceiving and comprised the study group, and 686 (38.7%) had non-surgical uterine evacuation (Figure 1).

The maternal demographic, obstetric and neonatal characteristics of the miscarriage pregnancy and the subsequent delivery, comparing the study and control groups, are presented in Table 1. The women in both groups had similar ages and obstetric histories. However, the women who underwent a D&C had a larger mean diameter gestational sac (24 ± 14.3 vs. 17.9 ± 9.4 mm, *p* < 0.01) and a larger fetus (6.1 ± 3.3 vs. 4.7 ± 3 weeks, *p* < 0.01) at the time of diagnosis. Among women in the D&C group, 108 women (9.9%) had pre-treatment with misoprostol, and in the non-surgical group, 558 women (81.3%) underwent uterine evacuation following misoprostol treatment (the remaining 128 women (18.7%) had a spontaneous miscarriage).

The D&C procedure was urgent in 215 of the cases (19.8%) and planned elective D&C in the rest. Cervical dilation was performed in 840 of the cases (77.8%). The miscarriage to pregnancy interval was comparable between the two groups (88.7 ± 44.4 vs. 86 ± 43.6 days, *p* = 0.20), as well as the rate of fertility treatments, multifetal gestation, hypertensive disorders of pregnancy and diabetes (pre-gestational and gestational).

The maternal obstetric and neonatal delivery outcomes, stratified by the mode of uterine evacuation in the prior pregnancy, are presented in Table 2.

### 3.1. Primary Outcome

Overall, 86 women had preterm delivery (4.9%). There was no difference in the PTD rate between the groups (58 (5.3%) vs. 28 (4.1%), *p* = 0.23).

### 3.2. Secondary Outcomes

No significant difference was observed between the two groups in all of the assessed maternal outcomes: mean gestational age at delivery, rates of premature rupture of membranes, placental abruptions, chorioamnionitis, cesarean deliveries (both overall and in-labor), retained placenta/placental fragments and postpartum hemorrhages. Regarding the neonatal outcomes, the mean neonatal birthweight was similar, and there was no significant difference in the rate of intrauterine fetal deaths, small-for-gestational-age newborns, Apgar scores and neonatal intensive care unit (NICU) admissions between the two groups. To neutralize the potential effect of multiple D&C on the maternal and neonatal outcomes, an additional analysis for women with only a single previous pregnancy loss was done (Table S1). No statistically significant differences were observed between the two study groups in all of the assessed outcomes.

An additional analysis was done comparing the maternal and neonatal adverse outcomes between four subgroups: (1) Expectant management (reference group), (2) Medical treatment, (3) Elective D&C and (4) Urgent D&C (Table S2). Again, no statistically significant differences were observed between the sub-groups in all of the assessed outcomes. However, due to the division of cases among the four groups and the relatively low case rates, the sample size is maybe too small to identify low to moderate levels of increased risk.

An adjusted multivariable logistic regression analysis for significant covariates and confounders to examine the association between D&C and PTD in the subsequent delivery revealed that D&C was not independently associated with PTD in the subsequent delivery (aOR 1.74 (0.89–3.40), *p* = 0.11) (Table 3).

## 4. Discussion

In this retrospective study, we analyzed data that were systematically collected and periodically validated regarding women who conceived within 6 months following a first trimester missed or incomplete abortion. The analysis was performed to reach conclusions regarding the potential adverse pregnancy outcomes within 6 months of uterine evacuation, comparing D&C and medical or expectant management. We found similar rates of any adverse maternal or neonatal outcomes between the study groups.

Based on this study’s univariate analysis results, the PTD rate was similar between the groups, and the maternal and neonatal obstetrics outcomes were similar. In an adjusted multivariable logistic regression analysis, we found that D&C was not independently associated with PTD in the subsequent delivery.

The matter of choosing the desired means of treating EPL has so far been approached mostly via the aspect of immediate outcomes for each management option, such as the need for unplanned or additional surgical evacuation, excessive bleeding and blood transfusion requirement [5,6,7,8].

Regarding subsequent obstetric outcomes, the studies performed so far have demonstrated inconsistent findings. While some found similar outcomes in comparing the methods of uterine evacuation, others found higher rates of PPH^13^, PTB and low birth weight (LBW) [13,14,17,18]. As in our study, Lemmers et al. followed women until the first new pregnancy subsequent to D&C or expectant management to assess the fertility and obstetric outcome. They reported similar rates of conception, ongoing pregnancy, PTB and CD [12].

On the other hand, Lohmann-Bigelow et al. compared the pregnancy outcomes of women following D&C to the overall expected incidences in the literature. They found similar rates of PTB, preeclampsia, placental abruption, malpresentation, cervical incompetence, first trimester bleeding and miscarriage but a significantly higher incidence of PPH^13^. In our current study, the D&C group had indeed more cases of PPH in the subsequent pregnancy, but it was, however, statistically insignificant (*p* = 0.08). This difference might be explained by the fact that the study that found this association included a relatively small sample size (*n* = 114 pregnancies), compared its result with previously recorded rates in the medical literature (there was no control group in this study) and did not exclude pregnancies conceived >6 months after the EPL.

Other studies found an association between D&C and subsequent PTB [15]. McCarthy et al. compared women with previous pregnancy loss (miscarriage or termination of pregnancy) with women with no previous pregnancy loss. They found that a single previous miscarriage or termination of pregnancy was not significantly associated with an increased risk of spontaneous PTB. In contrast, miscarriages treated with D&C demonstrated a significant association with spontaneous PTB.

Situ et al. examined the perinatal outcomes of the first pregnancy after a termination of pregnancy (TOP) by the method of previous TOP (medical vs. surgical) in a large nationwide register-based study and demonstrated an increased risk for both PTB and lower birth weight (LBW) [18]. Lemmers et al. (2016) provided a systematic review of twenty-one studies reporting on 1,853,017 women, comparing cases with a history of D&C for EPL or TOP and cases with no such background, and they found a higher risk for PTB [19]. Another systematic review and meta-analysis of 124,133 women from five studies was done by Saccone et al. and included women with spontaneous miscarriages. They have found that women with a prior D&C had a significantly higher risk of PTB compared with those who did not have that history [20].

Possible explanations for the difference between the findings of these studies and those of the current study might be the small sample size of McCarthy et. al’s study, the difference in the population that was studied (the TOP population may differ from the spontaneous miscarriage population) and the different methodologies used. In addition, these studies did not adjust for the time from the EPL to the subsequent pregnancy, and most of them did not report the rate of mechanical dilation within the D&C group.

An important factor that might explain the different findings regarding the obstetric outcomes following a miscarriage is the interpregnancy interval (IPI) from the miscarriage to the subsequent pregnancy. For instance, in a large study of 258,108 women from Latin America who had their previous pregnancy result in a miscarriage, the women with an IPI of less than 6 months had an increased risk of PTB, very PTB, low birth weight (LBW) and very LBW [21].

On the contrary, Love et al., in a population-based retrospective cohort study from Scotland, found that, compared with women with an interpregnancy interval of 6–12 months, women who conceived again within six months were less likely to have a CD, PTB or infant of LBW [22]. Wong et al. compared the obstetric outcomes between those with an IPI ≤ 3 months to those with an IPI of 3–6 months, 6–9 months, 9–12 months and >12 months and found similar rates of PTB, preeclampsia and Gestational Diabetes [23]. In a systematic review and meta-analysis of sixteen studies including 1,043,840 women, women with an IPI of less than 6 months had a lower risk of a further miscarriage and PTB and similar risks of stillbirth, low birthweight and pre-eclampsia. These findings were similar when an IPI of <6 months was compared with an IPI of 6–12 months and >12 months [24].

## 5. Strengths and Limitations

There are several notable strengths to this study: (1) It includes a large study population, including women meeting the qualifying criteria from a medical center at which 10% of all national deliveries are managed; (2) It is based on real-time data validation; (3) All of the costs of antenatal care, birth and postpartum care for mothers and children are uniformly covered by National Health Insurance for the entire study period; (4) All of the mother–child data included were taken from a singular hospital setting with no inter-hospital transfers, which may overcome a potential selection bias; (5) The departmental surgical protocols used in the D&C were similar throughout the study period; (6) The study includes only cases of first trimester spontaneous miscarriages and the obstetric outcomes of women who conceived within 6 months.

There are several limitations to this study: (1) The inherent limitation of our study’s retrospective design, which is based on data extracted from patients’ medical records, and, thus, some important information such as the type of previous pregnancy loss (surgical abortion, medical abortion, natural miscarriage, natural other losses) was missing and cannot be included in our multivariate analysis. Thus, it could be that, among women with previous multiple losses, the cumulative type of loss will be a factor for adverse outcome. (2) This is a single-center study, the population of which has specific characteristics, particularly pertaining to the motivation for a large family size. (3) As it is a surgical procedure, D&C outcomes might be dependent on the performing surgeon’s skills and experience. Pregnancies following D&Cs that were performed by other practitioners or in different medical centers may have different outcomes. This factor should be furthered explored in other studies. (4) As the study includes only cases of women who conceived within 6 months, the pregnancy outcomes of women who conceived beyond 6 months cannot be inferred from this study.

In conclusion, we found that, after excluding women with multiple miscarriages within six months of their first miscarriage, there were no significant differences in the maternal and neonatal obstetric outcomes for pregnancies occurring within six months of a first trimester pregnancy loss treated by either D&C or expectant management or medical treatment at a single medical center. Watchful waiting or the medical treatment of a first trimester missed abortion present similar risks as D&C to pregnancies conceived within six months of the index miscarriage. This was also true when a sub-group analysis for Elective D&C and Urgent D&C was done. However, due to the relatively low case rates, the sample size is maybe too small to identify low to moderate levels of increased risk.

Further studies to strengthen these findings should include repetition of the study design with larger data sets, consider adding the stratification of outcomes by the operating surgeon’s experience and perhaps add a comparison between different interpregnancy intervals.

## Figures and Tables

**Table 1:** Demographic and obstetric characteristics of the women stratified by the mode of uterine evacuation in the prior miscarriage.

	No D&C *n* = 686	D&C *n* = 1087	*p* Value
Maternal age, years	29.5 ± 5.8	29.2 ± 6	0.40
Miscarriage to pregnancy interval, days	86 ± 43.6	88.7 ± 44.4	0.20
Previous miscarriages	1.2 ± 0.6	1.2 ± 0.6	0.90
Miscarriage ≥ 3	31 (4.5%)	47 (4.3%)	0.85
Gravidity	5.2 ± 2.9	5.1 ± 2.8	0.40
Parity	4 ± 2.7	3.9 ± 2.6	0.38
Primipara	128 (18.7%)	219 (20.1%)	0.44
Number of previous CDs	0.2 ± 0.6	0.2 ± 0.6	0.77
Previous cesarean delivery, any	83 (12.1%)	139 (12.8%)	0.67
Fertility treatments	21 (3.1%)	40 (3.7%)	0.49
Hypertensive disorders of pregnancy	14 (2%)	20 (1.8%)	0.76
Diabetes (pre-gestational + gestational)	34 (5%)	53 (4.9%)	0.94
Obesity (BMI ≥ 30)	57 (14.3%)	115 (17.3%)	0.20
Multifetal gestation	11 (1.6%)	22 (2%)	0.52
Induction of labor	79 (12%)	113 (11%)	0.52

BMI—Body Mass Index. CD—Cesarean Delivery. Data are mean ± standard deviation; number (%).

**Table 2:** Maternal and neonatal obstetrics outcomes among the study groups.

	No D&C *n* = 686	D&C *n* = 1087	*p* Value
Gestational age at delivery	39.1 ± 2	39.2 ± 1.8	0.48
Gestational age at delivery < 37 week	28 (4.1%)	58 (5.3%)	0.23
Gestational age at delivery > 41 week	64 (9.3%)	92 (8.5%)	0.53
Premature rupture of membranes	87 (12.7%)	139 (12.8%)	0.95
Prolonged hospital stay *	13 (1.9%)	15 (1.4%)	0.40
Retained placenta/placental fragments	27 (4.2%)	32 (3.2%)	0.27
Maternal ICU admission	0 (0%)	1 (0.1%)	0.43
Postpartum hemorrhage	68 (9.9%)	82 (7.5%)	0.08
Placental abruption	16 (2.3%)	17 (1.6%)	0.24
Non-vertex presentation	20 (2.9%)	28 (2.6%)	0.67
Hemoglobin drop, g/dL	1.2 ± 1.1	1.2 ± 1	0.71
Hemoglobin drop ≥ 4 g/dL	26 (3.8%)	30 (2.8%)	0.23
Chorioamnionitis	14 (2%)	11 (1%)	0.07
Puerperal fever	9 (1.3%)	9 (0.8%)	0.32
Blood products transfusion	7 (1%)	7 (0.6%)	0.38
Hysterectomy	0 (0%)	0 (0%)	N/A
In labor cesarean	31 (4.5%)	48 (4.4%)	0.92
Cesarean delivery	65 (9.5%)	118 (10.9%)	0.35
Birthweight	3292.3 ± 502.5	3295 ± 503.5	0.91
Birthweight ≥ 4000 g	29 (4.2%)	45 (4.1%)	0.93
Large for gestational age	81 (11.8%)	126 (11.6%)	0.89
Small for gestational age	50 (7.3%)	73 (6.7%)	0.65
Intrauterine Fetal Death	4 (0.6%)	5 (0.5%)	0.72
1-Minute Apgar score < 7	29 (4.3%)	50 (4.6%)	0.72
5-Minute Apgar score < 7	17 (2.5%)	31 (2.9%)	0.64
NICU admission	43 (6.3%)	61 (5.6%)	0.56
Birth asphyxia	4 (0.6%)	9 (0.8%)	0.55

Data are mean ± standard deviation; number (%); D&C—Dilation and Curettage; ICU—Intensive Care Unit, NICU—Neonatal Intensive Care Unit. * prolonged postpartum hospital stays of >5 days for vaginal deliveries and >7 days for CD.

**Table 3:** Multivariate logistic regression analysis for the association between the mode of uterine evacuation and preterm delivery in the subsequent delivery (Adjusted Odds Ratio).

	*p* Value	aOR	95%CI	
Multifetal gestation	<0.01	29.67	12.58	69.97
Parity	0.01	0.62	0.44	0.88
Previous cesarean delivery, any	0.02	2.11	1.14	3.92
Gravidity	0.06	1.32	0.99	1.77
D&C	0.11	1.74	0.89	3.40
Fertility treatments	0.14	1.92	0.81	4.57
Cervical Dilation	0.16	0.63	0.33	1.19
Hypertensive disorders of pregnancy	0.32	2.00	0.51	7.76
Diabetes (pre-gestational + gestational)	0.49	1.40	0.54	3.62

CI—Confidence Interval; aOR—Adjusted Odds Ratio; D&C—Dilation and Curettage.

## Data Availability

Not applicable.

## Supplementary Materials

The following supporting information can be downloaded at: https://www.mdpi.com/article/10.3390/jcm11102767/s1, Table S1. Maternal and neonatal obstetrics outcomes among women with previous single pregnancy loss without and without D&C. Table S2. Maternal and neonatal obstetrics outcomes among women with different type of pregnancy loss management.
