# Supplementary material for: Maternal and Neonatal Outcomes of Women Conceived Less Than 6 Months after First Trimester Dilation and Curettage
Source: J Clin Med. 2022 May 13;11(10):2767. doi: 10.3390/jcm11102767 (PMC9147896; doi:10.3390/jcm11102767)
Supplement: Supplementary file 1 [file jcm-11-02767-s001.zip › jcm-1673181-supplementary.pdf]

Journal of
Clinical Medicine

MDPI

Table S1. Maternal and neonatal obstetrics outcomes among women with previous single pregnancy loss without and without D&C.

	No D&C *n* = 600	D&C *n* = 956	*p* Value
Gestational age at delivery	39.1±2	39.2±1.7	0.23
Gestational age at delivery < 37 week	26 (4.3%)	44 (4.6%)	0.80
Gestational age at delivery > 41 week	57 (9.5%)	83 (8.7%)	0.59
Premature rupture of membranes	79 (13.2%)	119 (12.4%)	0.68
Prolonged hospital stay *	12 (2%)	14 (1.5%)	0.42
Retained placenta/placental fragments	24 (4.3%)	30 (3.4%)	0.37
Maternal ICU admission	0 (0%)	1 (0.1%)	0.43
Postpartum hemorrhage	60 (10%)	73 (7.6%)	0.10
Placental abruption	15 (2.5%)	15 (1.6%)	0.19
Non-vertex presentation	16 (2.7%)	26 (2.7%)	0.95
Hemoglobin drop, gram/dL	1.3 ± 1.1	1.2 ± 1	0.64
Hemoglobin drop ≥ 4 gram/dL	23 (3.8%)	26 (2.7%)	0.22
Chorioamnionitis	11 (1.8%)	11 (1.2%)	0.27
Puerperal fever	8 (1.3%)	9 (0.9%)	0.47
Blood products transfusion	5 (0.8%)	6 (0.6%)	0.64
Hysterectomy	0 (0%)	0 (0%)	N/A
In labor cesarean	28 (4.7%)	40 (4.2%)	0.65
Cesarean delivery	55 (9.2%)	95 (9.9%)	0.62
Birthweight	3286.7 ± 493.6	3298.3 ± 483.8	0.65
Birthweight ≥ 4000 grams	21 (3.5%)	40 (4.2%)	0.50
Large for gestational age	64 (10.7%)	113 (11.8%)	0.48
Small for gestational age	45 (7.5%)	61 (6.4%)	0.40
Intrauterine Fetal Death	3 (0.5%)	3 (0.3%)	0.56
1-Minute Apgar score < 7	27 (4.5%)	44 (4.6%)	0.94
5-Minute Apgar score < 7	16 (2.7%)	27 (2.8%)	0.86
NICU admission	38 (6.4%)	52 (5.4%)	0.46
Birth asphyxia	3 (0.5%)	8 (0.8%)	0.44

Data are mean ± standard deviation; number (%); D&C–Dilation and Curettage ICU–Intensive Care Unit, NICU Neonatal intensive-care unit. * prolonged postpartum hospital stays of >5 days for vaginal deliveries and > 7 days for CD.

Table S2. Maternal and neonatal obstetrics outcomes among women with different type of pregnancy loss management.

	Expectant Management (Reference Group) *n* = 128	Medical *n* = 558	*p* Value	Elective D&C *n* = 872	*p* Value	Urgent D&C *n* = 215	*p* Value
Gestational age at delivery	39.3 ± 1.3	39.1 ± 2.2	0.42	39.2 ± 1.8	0.71	39.2 ± 1.7	0.69
Gestational age at delivery < 37 week	3 (2.3%)	25 (4.5%)	0.27	43 (4.9%)	0.19	15 (7%)	0.06
Gestational age at delivery > 41 week	16 (12.5%)	48 (8.6%)	0.17	77 (8.8%)	0.18	15 (7%)	0.08
Premature rupture of membranes	20 (15.6%)	67 (12%)	0.27	114 (13.1%)	0.43	25 (11.6%)	0.29
Prolonged hospital stay *	2 (1.6%)	11 (2%)	0.76	15 (1.7%)	0.90	0 (0%)	0.07
Retained placenta/placental fragments	7 (5.9%)	20 (3.8%)	0.30	25 (3.1%)	0.12	7 (3.5%)	0.31

Maternal ICU admission	0 (0%)	0 (0%)	N/A	1 (0.1%)	0.70	0 (0%)	N/A
Postpartum hemorrhage	9 (7%)	59 (10.6%)	0.23	65 (7.5%)	0.86	17 (7.9%)	0.77
Placental abruption	5 (3.9%)	11 (2%)	0.19	14 (1.6%)	0.08	3 (1.4%)	0.14
Non-vertex presentation	4 (3.1%)	16 (2.9%)	0.88	23 (2.6%)	0.75	5 (2.3%)	0.66
Hemoglobin drop, gram/dL	1.1 ± 1.1	1.3 ± 1.1	0.10	1.2 ± 1.1	0.24	1.3 ± 1	0.14
Hemoglobin drop ≥ 4 gram/dL	4 (3.1%)	22 (3.9%)	0.66	26 (3%)	0.93	4 (1.9%)	0.45
Chorioamnionitis	1 (0.8%)	13 (2.3%)	0.26	8 (0.9%)	0.88	3 (1.4%)	0.61
Puerperal fever	2 (1.6%)	7 (1.3%)	0.78	6 (0.7%)	0.30	3 (1.4%)	0.90
Blood products transfusion	0 (0%)	7 (1.3%)	0.20	5 (0.6%)	0.39	2 (0.9%)	0.28
Hysterectomy	0 (0%)	0 (0%)	N/A	0 (0%)	N/A	0 (0%)	N/A
In labor cesarean	5 (3.9%)	26 (4.7%)	0.71	36 (4.1%)	0.91	12 (5.6%)	0.49
Cesarean delivery	12 (9.4%)	53 (9.5%)	0.97	96 (11%)	0.58	22 (10.2%)	0.80
Birthweight	3332.4 ± 461.5	3283.1 ± 511.3	0.32	3292.5 ± 504.4	0.40	3305.3 ± 500.7	0.62
Birthweight ≥ 4000 grams	6 (4.7%)	23 (4.1%)	0.77	33 (3.8%)	0.62	12 (5.6%)	0.72
Large for gestational age	16 (12.5%)	65 (11.7%)	0.79	97 (11.1%)	0.65	29 (13.5%)	0.79
Small for gestational age	9 (7%)	41 (7.4%)	0.90	63 (7.2%)	0.93	10 (4.7%)	0.35
Intrauterine Fetal Death	1 (0.8%)	3 (0.5%)	0.74	4 (0.5%)	0.63	1 (0.5%)	0.71
1-Minute Apgar score < 7	6 (4.7%)	23 (4.2%)	0.79	37 (4.3%)	0.83	13 (6.1%)	0.59
5-Minute Apgar score < 7	3 (2.3%)	14 (2.5%)	0.90	25 (2.9%)	0.73	6 (2.8%)	0.80
NICU admission	7 (5.5%)	36 (6.5%)	0.67	46 (5.3%)	0.93	15 (7%)	0.58
Birth asphyxia	0 (0%)	4 (0.7%)	0.34	8 (0.9%)	0.28	1 (0.5%)	0.44

Data are mean ± standard deviation; number (%); D&C–Dilation and Curettage ICU–Intensive Care Unit, NICU Neonatal intensive-care unit. * prolonged postpartum hospital stays of >5 days for vaginal deliveries and > 7 days for CD.
